# Biomarker discovery and development of prognostic prediction model using metabolomic panel in breast cancer patients: a hybrid methodology integrating machine learning and explainable artificial intelligence

**DOI:** 10.3389/fmolb.2024.1426964

**Published:** 2024-12-18

**Authors:** Fatma Hilal Yagin, Yasin Gormez, Fahaid Al-Hashem, Irshad Ahmad, Fuzail Ahmad, Luca Paolo Ardigò

**Affiliations:** ^1^ Department of Biostatistics and Medical Informatics, Faculty of Medicine, Inonu University, Malatya, Türkiye; ^2^ Department of Management Information Systems, Faculty of Economics and Administrative Sciences, Sivas Cumhuriyet University, Sivas, Türkiye; ^3^ Department of Physiology, College of Medicine, King Khalid University, Abha, Saudi Arabia; ^4^ Department of Medical Rehabilitation Sciences, College of Applied Medical Sciences, King Khalid University, Abha, Saudi Arabia; ^5^ Respiratory Care Department, College of Applied Sciences, Almareefa University, Riyadh, Saudi Arabia; ^6^ Department of Teacher Education, NLA University College, Oslo, Norway

**Keywords:** breast cancer, metabolomics, feature selection, explainable artificial intelligence, prognostic model

## Abstract

**Background:**

Breast cancer (BC) is a significant cause of morbidity and mortality in women. Although the important role of metabolism in the molecular pathogenesis of BC is known, there is still a need for robust metabolomic biomarkers and predictive models that will enable the detection and prognosis of BC. This study aims to identify targeted metabolomic biomarker candidates based on explainable artificial intelligence (XAI) for the specific detection of BC.

**Methods:**

Data obtained after targeted metabolomics analyses using plasma samples from BC patients (n = 102) and healthy controls (n = 99) were used. Machine learning (ML) models based on raw data were developed, then feature selection methods were applied, and the results were compared. SHapley Additive exPlanations (SHAP), an XAI method, was used to clinically explain the decisions of the optimal model in BC prediction.

**Results:**

The results revealed that variable selection increased the performance of ML models in BC classification, and the optimal model was obtained with the logistic regression (LR) classifier after support vector machine (SVM)-SHAP-based feature selection. SHAP annotations of the LR model revealed that Leucine, isoleucine, L-alloisoleucine, norleucine, and homoserine acids were the most important potential BC diagnostic biomarkers. Combining the identified metabolite markers provided robust BC classification measures with precision, recall, and specificity of 89.50%, 88.38%, and 83.67%, respectively.

**Conclusion:**

In conclusion, this study adds valuable information to the discovery of BC biomarkers and underscores the potential of targeted metabolomics-based diagnostic advances in the management of BC.

## 1 Introduction

The GLOBOCAN 2020 report highlights breast cancer (BC) as the global cancer with the highest incidence worldwide (Rahimzadeh et al., 2021). This report is underscored by its alarming mortality rates, accounting for 685,000 deaths in 2020 and establishing it as the foremost cause of cancer-related mortality in women. Representing 11% of all cancer cases, breast cancer diagnoses in women totaled approximately 2.3 million. A notable escalation of 20% in prevalence and a 14% increase in global mortality rates have been observed since the 2008 report (Kashyap et al., 2022).

Projections from global breast cancer surveillance studies indicate a looming 40% rise in new cases, surpassing three million, with a simultaneous 50% surge in breast cancer-related deaths, reaching one million by 2040 (Arnold et al., 2022). In addition, BC increases female deaths significantly in underdeveloped countries, ranks fifth overall in cancer-related deaths, and is the primary cause of death among women in these regions. In developed countries, breast cancer ranks second after lung cancer in cancer-related deaths (Cao et al., 2021).

While the average 5-year survival rate for BC at all stages hovers around 90% (Siegel et al., 2018), the 5-year survival rate significantly rises to nearly 99% for early-stage BC (comprising stages I and II). Unfortunately, only 61% of patients receive an early-stage diagnosis (DeSantis et al., 2017). Despite the demonstrated high sensitivity (>90%) of various clinical and experimental imaging techniques in diagnosing symptomatic BC patients, there remains a conspicuous absence of an effective diagnostic tool for early-stage BC (EBC) detection (Fiorica, 2016; Bonilla et al., 2017). Consequently, there is an urgent demand for a detection method that ensures precise and early diagnosis and screening, ultimately facilitating prompt treatment and enhancing overall BC survival rates.

Examining metabolites within biological pathways offers crucial insights into the functional phenotype of tumors. The exploration of metabolic alterations in cancer cells dates back to the first half of the 20th century, notably with Otto Warburg’s discovery of aerobic glycolysis, now recognized as the Warburg effect, a prevalent feature in various tumors (Tenori et al., 2015; Schwartz et al., 2017). Furthermore, metabolites contribute essential information that intersects with fields such as proteomics, transcriptomics, and genomics, enhancing our systematic understanding of biological systems.

Although numerous studies have shown promise in investigating the metabolomic profile of BC, these findings require additional validation before integration into clinical practice (Tenori et al., 2015; Jobard et al., 2014; Tenori et al., 2012; Oakman et al., 2011; Wei et al., 2013; Hart et al., 2017) Moreover, existing research lacks an exploration of metabolomic biomarkers for BC through the lens of explainable artificial intelligence (XAI).

Given the potential of the metabolomic approach and the imperative for more comprehensive metabolomic investigations in BC, our study seeks to identify potential biomarkers for early BC detection. Additionally, we aim to develop an interpretable prediction model using XAI to enhance the understanding of metabolomic biomarkers in the context of BC.

## 2 Materials and methods

### 2.1 Dataset

This research utilized publicly available (open-access) targeted metabolomics panel data to differentiate BC patients and healthy controls (Jasbi et al., 2019). The study was conducted according to the principles of the Declaration of Helsinki and was approved by the Inonu University Health Sciences Non-Interventional Clinical Research Ethics Committee (protocol code = 2024/5750). A total of 201 samples were included in the study, collected from individuals who underwent an overnight fast; among them, 102 were BC patients and 99 were healthy controls. Controls were matched with BC patients based on age. The study aimed to identify potential metabolic biomarkers through feature selection and explainable artificial intelligence techniques, in addition to the development of the ML prediction model that could increase sensitivity and specificity in detecting the early stage of BC. A targeted liquid chromatography-tandem mass spectrometry (LC-MS/MS) approach was used to examine the metabolomic panel. Further information regarding sample preparation and the conditions of liquid chromatography and mass spectrometry can be found in the Supplementary Material (Jasbi et al., 2019).

### 2.2 Methods

Thousands In our study, ML methods were proposed for the early diagnosis of BC. The SHapley Additive exPlanations (SHAP) method was utilized to explain these proposed methods and to attempt the determination of features effective in early BC diagnosis. In this manner, SHAP values generated from the results of classification algorithms were employed for feature selection. The hyper-parameter optimization of the proposed models was carried out using the Random Search (RS) method within the context of nested cross-validation (nestedCV). In addition to SHAP, RS, and nestedCV methods, classification algorithms such as Random Forest (RF), Gradient Boosted (GB), Logistic Regression (LR), and Support Vector Machines (SVM) were employed in our study.

### 2.3 Random forest

The RF algorithm is a popular ensemble learning technique extensively employed in machine learning. It operates by amalgamating numerous decision trees to construct a robust predictive model. In an RF model, each decision tree is developed by training on a subset of data, where different features are assessed against varying examples. Subsequently, every tree generates its individual predictions, and the collective prediction is determined based on the average of these forecasts or the majority vote among the classes. RF exhibits resilience against overfitting and tends to exhibit superior performance particularly on extensive datasets. Additionally, its utility extends to discerning the significance of variables owing to its capability to gauge the relative importance levels among features (Breiman, 2001; Yagin et al., 2024).

### 2.4 Gradient boosted

GB stands as a robust ML algorithm employed in constructing predictive models. It achieves this by amalgamating various decision trees, culminating in a potent predictive model. Each tree undergoes sequential training, with a focus on rectifying errors that its predecessor failed to address. Leveraging this sequential structure, GB iteratively integrates new trees into training with the aim of error minimization. The efficacy of GB is contingent upon meticulous parameter adjustments; otherwise, it may lead to overfitting. Hence, its application warrants careful consideration to attain a well-balanced model (Yagin B. et al., 2023; Yagin F. H. et al., 2023).

### 2.5 Logistic regression

LR, despite its name implying regression, serves as a ML algorithm predominantly employed in classification tasks. Its primary function is to discriminate between two or more classes, typically presenting its output in the form of a probability value. Operating on a linear equation, LR models the correlation between input features and output classes, with the equation’s outcome transformed into a classification decision through a logistic function. Throughout the training process, it employs optimization techniques such as the maximum likelihood method or gradient descent to ascertain the optimal parameters. Despite its advantages in simplicity, interpretability, and swiftness, the efficacy of LR might diminish in scenarios where it fails to discern linear boundaries, encountering difficulty in capturing intricate relationships (Khandezamin et al., 2020; Donisi et al., 2022; Dedeturk and Akay, 2020).

### 2.6 Support vector machines

SVMs represent a potent ML algorithm extensively applied in classification tasks. SVMs aim to delineate a decision boundary between two classes and position data points optimally within these bounds. The hallmark of SVMs lies in their ability to generalize beyond the dataset, ensuring data points are segregated by the widest possible margin along the decision boundary. This algorithm operates by leveraging support vectors, which effectively facilitate the clearest separation between distinct classes. Moreover, SVMs exhibit efficacy in handling multidimensional datasets and possess the capability to segregate datasets that lack linear separability by mapping them onto high-er-dimensional spaces. Nevertheless, while dealing with sizable datasets, SVMs encounter heightened computational complexity, demanding precise parameter settings (Sheykhmousa et al., 2020; Guo et al., 2021; Li and Sun, 2020).

### 2.7 Nested cross validation

nestedCV is a cross-validation technique employed to robustly assess the performance of a model. This approach integrates a two-tiered cross-validation framework. The outer loop segregates the dataset into training and testing folds, while the inner cross-validation structure is applied within each training fold. Within the inner loop, each training fold is further divided into smaller folds, utilized for hyper-parameter tuning or selection of the model. Consequently, a model trained with optimal hyper-parameters is derived for each test fold in the outer loop, and an aggregated performance metric is computed by consolidating performance evaluations. This method finds extensive application in reliably conducting both hyper-parameter optimization and performance evaluations of the model (Wainer and Cawley, 2021; Yates et al., 2023; Hosseini et al., 2020).

### 2.8 Random search hyper-parameter optimization

RS hyper-parameter optimization is a technique utilized to enhance the performance of ML models by optimizing hyper-parameters. This method involves a random selection of values across hyper-parameters impacting the model’s performance. These selections are made randomly throughout the potential value distributions within a given hyper-parameter space. Through repetitive training and testing iterations using these randomly chosen hyper-parameter values, the objective is to maximize model performance or minimize error rates. RS often proves more efficient than Grid Search as it achieves favorable outcomes by making fewer attempts via random selections, avoiding exhaustive exploration of the entire hyper-parameter space. Particularly effective with expansive hyper-parameter domains, it reduces time investment, offering a more cost-effective approach. Widely favored for its adaptability and time-saving attributes, this method is widely preferred in machine learning applications (Bergstra and Bengio, 2012; Villalobos-Arias and Quesada-López, 2021; Bhat et al., 2018).

### 2.9 SHapley additive exPlanations

SHAP stands as an explainability technique utilized to elucidate the predictions of ML models and comprehend feature contributions. SHAP leverages Shapley values to assess the impact of individual features on predictions, aggregating these contributions to form the model’s output. Inspired by coalition games, Shapley values are employed to quantify each feature’s influence on predictions, considering their interactions with other features. This method furnishes an explanation value for each feature, aiding in discerning the significance of features in understanding the model’s predictions. Noteworthy is SHAP’s effectiveness in handling large datasets and complex models, rendering the model’s inner workings more comprehensible. Despite the additional computational costs associated with SHAP, its interpretability advantages and compliance with XAI principles make it a robust option for biomarker discovery in this study. By leveraging SHAP’s ability to provide a detailed breakdown of feature effects, we aim to increase the transparency, reliability, and clinical relevance of our predictive model for breast cancer diagnosis (Lundberg et al., 2020; Bifarin, 2023; Zheng et al., 2023).

### 2.10 Proposed approach

**FIGURE 1:**
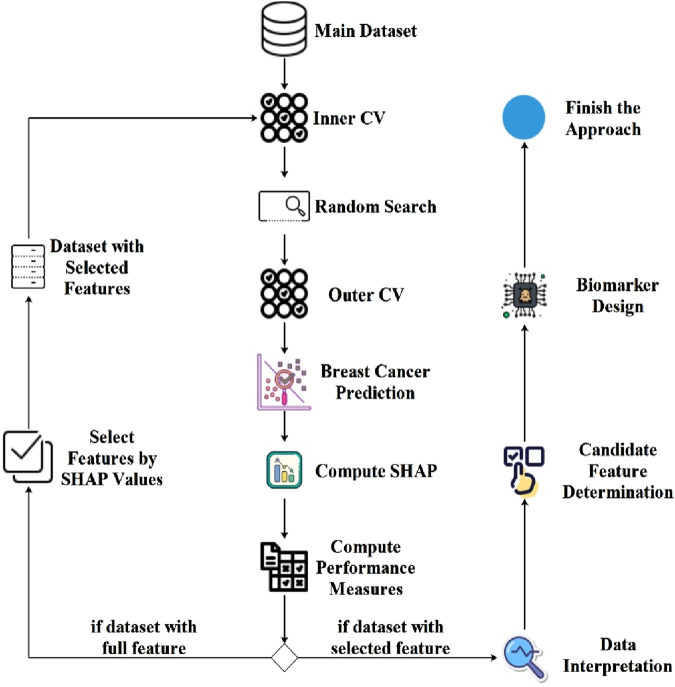
Flowchart diagram of the proposed approach generated using nestedCV, random search and SHAP computation.

In the initial phase of the proposed approach, the objective is to conduct hyper-parameter optimization and compute SHAP values utilizing the nestedCV method coupled with the random search method for EBC diagnosis. Subsequently, the second stage involves employing the SHAP values obtained in the first stage for feature selection, followed by repeating the training procedures from the initial phase. The flowchart illustrating the proposed approach is depicted in Figure 1.

As shown in Figure 1, the proposed model employs two different segmentation strategies: Inner CV and Outer CV. The Inner CV is used to perform hyper-parameter optimization and select the most suitable model. In this step, a 5-fold cross-validation is applied to each training fold of the Outer CV. On the other hand, the Outer CV is designed to assess the overall performance of the model and estimate its generalization error. For this purpose, a 10-fold cross-validation is performed on the entire dataset. Figure 1 demonstrates that within the proposed system, an initial step involves internal cross-validation to conduct hyper-parameter optimization through random search. Following this phase, model training proceeds using Outer CV, accompanied by the computation of SHAP values for the models. Subsequently, model performance metrics are computed based on the specified criteria. Should the model training processes utilize the complete set of features, feature selection is then implemented employing SHAP values, resulting in the creation of a new dataset. The finalization of the proposed approach entails reiteration of hyper-parameter optimization and training processes utilizing the newly generated dataset. Subsequently, the most significant features identified using the SHAP method were analyzed in the context of breast cancer diagnosis. Following this interpretative analysis, candidate features were selected for biomarker development, culminating in the final design of the biomarker.

### 2.11 Statistical analyses

The conformity of variables to normal distribution was examined using visual (histogram and probability graphs) and analytical (Shapiro-Wilk Test) methods. Descriptive statistics were expressed as mean ± standard deviation for normally distributed variables. Frequency (n) and percentage (%) values were calculated for qualitative variables. Statistical analyses were performed using SPSS 28.0 (IBM Corp., Armonk, NY, United States) package program.

## 3 Results

### 3.1 Univariate statistical analyses

Table 1 presents the results regarding the patients’ clinical data. The average age of patients in the BC group was higher at 54.637 ± 10.384 years compared to 51.556 ± 12.334 years in the control group. Among the BC patients, the distribution of menopausal status was as follows: 42.16% (43/102) were premenopausal, 5.88% (6/102) were perimenopausal, and the majority, 51.96% (53/102), were postmenopausal. Tumor laterality was predominantly observed in the right breast (53.92%, 55/102), followed by the left breast (42.16%, 43/102), with 3.92% (4/102) of cases involving bilateral tumors. The distribution of cancer stages showed that 23.53% (24/102) of patients were diagnosed at Stage I, 41.18% (42/102) at Stage II, and 35.29% (36/102) at Stage III. Regarding hormone receptor status, 93.14% (95/102) of patients were estrogen receptor (ER) positive, while only 6.86% (7/102) were ER negative. Progesterone receptor (PR) positivity was observed in 86.27% (88/102) of patients, with 13.73% (14/102) being PR negative. HER2 status was negative in 67.65% (69/102) of patients, while 12.75% (13/102) were borderline/intermediate, and 19.61% (20/102) were HER2 positive. Subtype distribution revealed that the majority of patients were ER/PR + HER2- (63.73%, 65/102), followed by ER/PR + HER2+ (17.65%, 18/102), ER/PR- HER2- (3.92%, 4/102), and ER/PR- HER2+ (1.96%, 2/102). The subtype was unknown in 12.75% (13/102) of cases. Furthermore, ductal carcinoma was the most common histological type, present in 90.20% (92/102) of cases, while lobular carcinoma and mixed ductal/lobular carcinoma accounted for 7.84% (8/102) and 1.96% (2/102) (Table 1).

**TABLE 1:** Data analysis on patients’ clinical information.

		Group	
		Control	BC
		Value	Value
Age*		51.556 ± 12.334	54.637 ± 10.384
Menopause Status**	pre		43 (42.16%)
	peri		6 (5.88%)
	post		53 (51.96%)
Side**	left		43 (42.16%)
	right		55 (53.92%)
	both		4 (3.92%)
Cancer stage**	I		24 (23.53%)
	II		42 (41.18%)
	III		36 (35.29%)
ER_**	-		7 (6.86%)
	+		95 (93.14%)
PR_**	-		14 (13.73%)
	+		88 (86.27%)
Her2**	-		69 (67.65%)
	borderline(2+)/intermediate		13 (12.75%)
	+		20 (19.61%)
Sub type**	ER/PR+ Her2+		18 (17.65%)
	ER/PR+ Her2-		65 (63.73%)
	ER/PR- Her2+		2 (1.96%)
	ER/PR- Her2-		4 (3.92%)
	Unknown		13 (12.75%)
Cancer type**	Ductal		92 (90.20%)
	Lobular		8 (7.84%)
	both		2 (1.96%)

BC, breast cancer; *mean ± standard deviation; **n (%).

### 3.2 Model preparation and hyper-parameter optimization

In our study, the proposed approach was implemented using a dataset comprising 201 clinical records, encompassing 102 BC patients and 99 control. The initial experimental phase involved developing the classification and hyper-parameter optimization stages of the proposed model utilizing Python’s sklearn library, specifically leveraging the RandomizedSearchCV, StratifiedKFold, and cross_val_score classes. StratifiedKFold was employed to ensure a balanced representation of samples across various classes within the folds. For inner cross-validation, a 5-fold setup was adopted, whereas 10 folds were utilized for outer cross-validation. The RandomizedSearchCV library facilitates random selections within the provided hyper-parameter space and conducts a set number of trials to identify the hyper-parameter configuration that yields optimal results. Within the proposed approach, the RandomizedSearchCV library was called 20 times. Adhering to the nestedCV paradigm, for each externally created fold, an internal cross-validation was executed with five folds to attain optimal values. The classification phase of the proposed model encompassed the utilization of four distinct classification methods: RF, GB, LR, and SVM. The classification models were developed using sklearn library of Python language. Table 2 delineates the optimized hyper-parameters for each method, the respective hyper-parameter spaces, and the resultant optimal values obtained for each fold.

**TABLE 2:** Hyper-parameter space information and optimum hyper-parameter values for the pro-posed models.

Model name	Hyper-parameter name	Hyper-parameter space	Optimum hyper-parameters for each fold
Random Forest	n_estimators	50, 100, 150, 200, 250, 300, 350, 400, 450, 500, 550	300, 400, 50, 200, 500, 50, 100, 350, 150, 300
	max_depth	2, 5, 10, 15, 20	10, 6, 4, 8, 6, 8, 12, 8, 4, 4
	min_samples_split	2, 4, 6, 8, 10, 12, 14, 16, 18, 20	15, 5, 20, 20, 15, 15, 20, 20, 5, 15
Gradient Boosting	n_estimators	50, 100, 150, 200, 250, 300, 350, 400, 450, 500, 550	300, 500, 500, 400, 400, 450, 200, 300, 250, 400
	max_depth	2, 5, 10, 15, 20	5, 2, 5, 2, 15, 5, 2, 2, 5, 2
	learning_rate	i/10 for i in range(1, 31, 1)	0.8, 1.1, 1.3, 1.3, 1.5, 0.6, 0.3, 0.7, 0.8, 0.6
Logistic Regression	C	2^−5^, 2^−4^, 2^−3^, 2^−2^, 2^−1^, 2^0^, 2^1^, 2^2^, 2^3^, 2^4^, 2^5^	0.625, 16, 1, 8, 8, 0.0625, 4, 0.03125, 8, 1
	max_iter	i for i in range (100, 1,501, 50)	100, 150, 300, 100, 150, 100, 500, 150, 150, 100
Support Vector Machine	C	2^−5^, 2^−4^, 2^−3^, 2^−2^, 2^−1^, 2^0^, 2^1^, 2^2^, 2^3^, 2^4^, 2^5^	8, 8, 32, 8, 16, 32, 4, 32, 16, 16
	max_iter	i for i in range (100, 1,501, 50)	1,500, 300, 150, 550, 1,350, 350, 300, 250, 750, 1,150

The function represented in Table 2 returned values within specified ranges by taking the starting, ending, and increment values of the specified range function, respectively. The “i” value employed in this function denoted each element within the list generated by the range function. Column “Optimum Hyper-Parameters for Each Fold” in Table 2 showcased the optimal values obtained for each external cross-validation, respectively.

### 3.3 Classification using all metabolite features

In the subsequent phase of the experiment, the performance metrics of the proposed classification methods were computed utilizing the acquired optimal hyper-parameters. In this context, accuracy, precision, recall, and specificity values for each fold were computed for each method. To gauge the model’s robustness, the standard deviation (std) of the performance values across folds was also determined. Table 3 shows the interfold std values for each metric and the average of the metric scores obtained in each fold.

**TABLE 3:** Performance values of proposed models calculated on the testing dataset.

Model name	Accuracy	Std of accuracy	Precision	Std of precision	Recall	Std of recall	Specificity	Std of specificity
RF	84.50%	11.05	88.29%	7.62	82.22%	11.32	70.44%	21.79
LR	82.52%	10.80	83.52%	10.64	82.36%	10.97	79.55%	16.11
GB	84.50%	10.82	86.72%	9.06	82.22%	11.10	79.44%	21.81
SVM	84.50%	11.50	87.46%	8.81	**84.26%**	11.80	71.44%	21.52

According to the findings presented in Table 3, RF, GB, and SVM exhibited the highest accuracy. However, when considering robustness based on the standard deviation of accuracy across each fold, LR emerged as the most robust method. Among the three high-scoring methods, GB was deemed the most robust. Regarding precision scores, RF not only attained the highest metric score but also demonstrated the most resilience. SVM emerged as the most successful method based on recall value, whereas LR displayed the greatest robustness. In terms of specificity, LR showcased both the highest performance and robustness. Upon comprehensive evaluation, all classification algorithms showed their weakest metric performance in specificity, with LR consistently displaying the most robustness. However, an overall assessment indicates no significant superiority of any classification method over the others.

### 3.4 Computing SHAP values and metabolite feature selection

**FIGURE 2:**
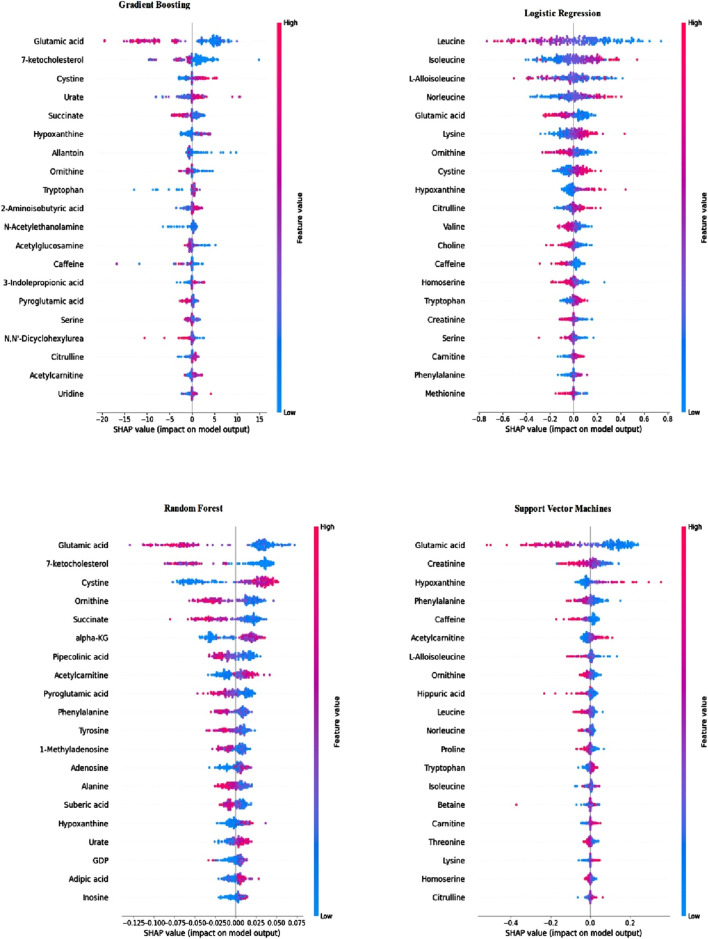
Feature importance of classification models calculated using SHAP values.

For the classification models, subsequent to the measurement of metric values, SHAP values were computed utilizing the SHAP Library in Python to gauge the feature impact during the training of each classification model. The importance of metabolite features, as per the obtained SHAP values, is separately listed for each classification. Figure 2 illustrates the importance of the top 20 metabolite features calculated using the SHAP method for each classification model.

As depicted in Figure 2, the metabolite importance order varies based on the classification method employed. Notably, according to the SHAP values calculated using trained RF, SVM, and GB models, the feature “glutamic acid” holds the utmost importance for subjectivity. In the case of the trained LR model, “glutamic acid” ranks as the fifth most critical metabolite for BC. Additionally, across all classifications, “glutamic acid,” “hypo-xanthine,” and “ornithine” consistently rank among the top 20 metabolites. Leveraging the data from Figure 2, metabolite biomarker selection was conducted individually, resulting in the identification of the top 20 metabolites for each trained model. The selected metabolite names are detailed in Table 4.

**TABLE 4:** Metabolite names that selected using SHAP values computed by trained classification models.

Model name	Selected metabolite names
RF	1-Methyladenosine, 7-ketocholesterol, Acetylcarnitine, Adenosine, Alanine, Cystine, GDP, Glutamic acid, Hypoxanthine, Inosine, Ornithine, Phenylalanine, Pipecolinic acid, Pyroglutamic acid, Tyrosine, Adipic acid, Suberic acid, Succinate, Urate, alpha-KG
LR	Caffeine, Carnitine, Choline, Citrulline, Creatinine, Cystine, Glutamic acid, Homoserine, Hypoxanthine, Isoleucine, L-Alloisoleucine, Leucine, Lysine, Methionine, Norleucine, Ornithine, Phenylalanine, Serine, Tryptophan, Valine
GB	2-Aminoisobutyric acid, 3-Indolepropionic acid, 7-ketocholesterol, Acetylcarnitine, Acetylglucosamine, Caffeine, Citrulline, Cystine, Glutamic acid, Hypoxanthine, N,N′-Dicyclohexylurea, N-Acetylethanolamine, Ornithine, Pyroglutamic acid, Serine, Tryptophan, Uridine, Allantoin, Succinate, Urate
SVM	Acetylcarnitine, Betaine, Caffeine, Carnitine, Citrulline, Creatinine, Glutamic acid, Hippuric acid, Homoserine, Hypoxanthine, Isoleucine, L-Alloisoleucine, Leucine, Lysine, Norleucine, Ornithine, Phenylalanine, Proline, Threonine, Tryptophan

### 3.5 Classification using selected metabolite features

Following the metabolite feature selection based on SHAP values derived separately from each trained RF, LR, GB, and SVM model, the classification models underwent retraining, and subsequent performance scores were computed. At this stage, rather than employing traditional feature selection methods, we utilized the explanations provided by the trained models to identify the most influential features. In this context, SHAP values were calculated separately for each trained model, and the features with the highest SHAP values were selected for inclusion in the new model. Thus, the SHAP method served a dual purpose in this study, facilitating both feature selection and model interpretability. Similar to the initial stage, the nestedCV method was employed, with five inner folds and 10 outer folds. Model hyper-parameters were optimized utilizing spaces in the models, encompassing all metabolites. Table 5 shows the interfold std values for each metric and the average of the metric scores obtained in each fold for models trained using selected metabolite features.

**TABLE 5:** Performance values of proposed models calculated on the testing dataset.

Trained model for feature selection	Classification model	Accuracy	Std of accuracy	Precision	Std of precision	Recall	Std of recall	Specificity	Std of specificity
RF	RF	86.02%	10.93	90.25%	6.33	85.77%	11.25	72.44%	23.44
	LR	85.07%	8.95	86.98%	7.82	84.91%	9.1	80.56%	18.15
	GB	83.52%	8.11	85.30%	7.09	83.37%	8.34	77.56%	18.81
	SVM	85.00%	10	89.15%	6.04	84.78%	10.13	70.56%	20.51
LR	RF	83.02%	10.8	85.93%	7.98	82.81%	11.12	71.44%	22.43
	LR	82.52%	8.77	83.78%	8.53	82.32%	8.89	77.56%	14.62
	GB	80.52%	9.9	83.19%	9.56	80.36%	10.23	75.44%	19.38
	SVM	84.02%	8.63	88.06%	5.49	83.77%	8.99	70.44%	19.37
GB	RF	87.50%	12.09	90.22%	9.69	87.22%	12.41	75.44%	22.71
	LR	83.07%	11.24	84.19%	10.89	82.96%	11.4	81.56%	18.9
	GB	83.55%	9.27	85.06%	8.61	83.31%	9.59	79.44%	19.39
	SVM	84.50%	10.11	88.86%	5.94	84.22%	10.4	69.44%	21.1
SVM	RF	84.02%	12.02	87.22%	9.04	83.77%	12.28	73.44%	24.36
	LR	88.52%	6.75	89.50%	6.79	88.38%	6.88	83.67%	10.69
	GB	80.60%	8.5	82.88%	7.44	80.45%	8.82	76.44%	19.84
	SVM	84.50%	10.59	87.01%	8.57	84.27%	10.93	72.44%	19.73

**FIGURE 3:**
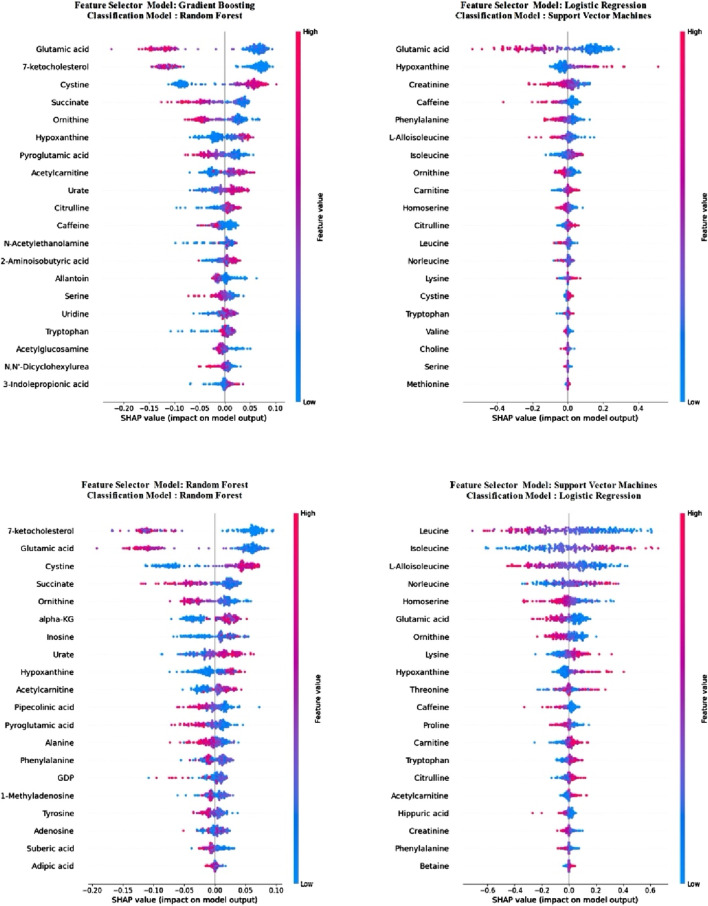
Metabolite importance of classification models calculated using SHAP values after feature selection.

Upon examining the results presented in Table 4, it is evident that the LR model, trained with selected metabolite features derived from the SHAP values calculated from the trained SVM model, outperforms other models across all metrics, excluding precision. Notably, this model also exhibits the lowest std values for these metrics. Comparatively, when contrasted with models utilizing all metabolites, the selection of metabolites using SHAP values appears to enhance model performance. To explore potential changes in metabolite importance when only the 20 selected metabolite features were employed, SHAP values were recalculated in the newly trained models, elucidating the models. Consequently, models trained in the initial stage, utilizing all metabolites, were also explained utilizing the SHAP method, doubling as the feature selection method. Figure 3 illustrates metabolite importance graphs for each classification utilized as a feature se-lection method in the first stage, alongside the model exhibiting the highest accuracy in the second stage. Specifically, the importance graphs for the RF, SVM, RF, and LR models trained in the second stage were sequentially drawn for the RF, LR, GB, and SVM models, respectively, trained using all metabolite features in the first stage.

**FIGURE 4:**
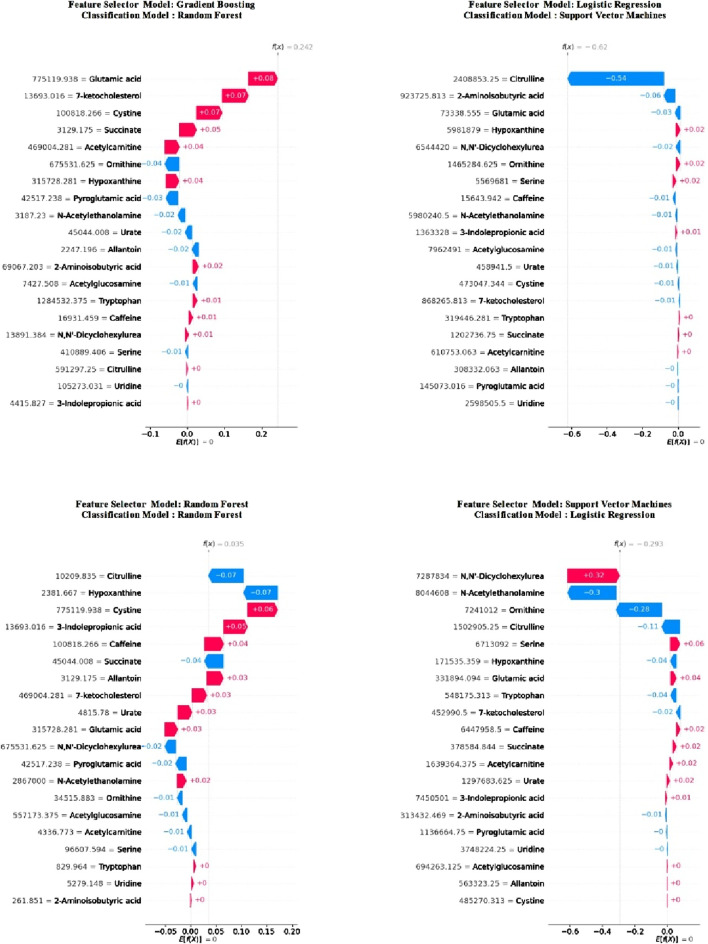
Waterfall diagram of classification models calculated using SHAP values after feature selection.

In Figure 3, following the feature selection, it is evident that the importance of metabolite features alters within the trained models. Notably, despite this variation, “glutamic acid” retained its status as the most crucial metabolites across both models. Moreover, it emerged as the second most importance metabolite in the RF model and ranked sixth in importance in LR. Among the other two metabolites common to all four models, “hypoxanthine” consistently ranked ninth at worst in all models, while “ornithine” held the seventh position in the least favourable scenario across all models. Detailed examination of the model explanations using SHAP is crucial. Therefore, SHAP waterfall plots for a selected positive example are presented separately for each model in Figure 4.

Upon examining these graphs, it is evident that the “glutamic acid” feature holds significant importance across all models. This finding aligns with the previously obtained summary SHAP plots, reinforcing the assumption that this feature plays a crucial role in model training.

In our study, to further investigate the explanations provided by SHAP, we examined the correlation between the selected features. For this purpose, Spearman Correlation coefficients were calculated between each pair of features. A correlation coefficient between −0.75 and 0.75 was considered to indicate no significant correlation, while values between −0.75 and −1 or 0.75 and 1 were interpreted as indicating a significant correlation. The features identified as having a significant correlation based on these calculations are presented in Table 6.

**TABLE 6:** Correlations between selected attributes according to SHAP results obtained from trained Machine Learning models.

Model name	Correlated feature 1	Correlated feature 2
LR	Isoleucine	L-Alloisoleucine
LR	Isoleucine	Leucine
LR	Isoleucine	Norleucine
LR	L-Alloisoleucine	Leucine
LR	L-Alloisoleucine	Norleucine
LR	Leucine	Norleucine
RF	Pipecolinic acid	Pyroglutamic acid
RF	Adipic acid	alpha-KG
SVM	Homoserine	Threonine
SVM	Isoleucine	L-Alloisoleucine
SVM	Isoleucine	Leucine
SVM	Isoleucine	Norleucine
SVM	L-Alloisoleucine	Leucine
SVM	L-Alloisoleucine	Norleucine
SVM	Leucine	Norleucine

Based on the results of the correlation analysis, it was observed that there was no correlation among any of the features selected using SHAP values from the GB model. In contrast, there was a correlation between two pairs of features selected using SHAP values from the RF model, a correlation between six pairs of features selected using SHAP values from the RF LR model, and a correlation between seven pairs of features selected using SHAP values from the RF model.

## 4 Discussion

There are some studies in the literature that describe biomarkers for BC detection using targeted metabolomics technology, an emerging field with significant diagnostic potential. However, these studies, using slightly different methodologies, have yielded different sets of biomarkers, creating diagnostic challenges in clinical practice. Additionally, some studies have produced predictive prognostic models after biomarker discovery, while others have not.

The current study used open access data analysing plasma samples from BC patients (102 cases) and healthy controls (99 cases) by targeted LC-MS/MS and found that identification of biomarker metabolites/features in BC enables accurate BC classification. The results of the study contribute to the existing literature by developing sensitive BC biomarkers and a more comprehensive metabolomic understanding of the pathogenic profile of BC.

In this study, SHAP was used as a feature selection tool due to its unique additive nature and interpretive powers in high-risk, clinical prediction modeling. SHAP values provide a consistent and mathematically robust approach to quantify feature importance by assigning an importance value to each feature according to its contribution to model predictions, based on Shapley values from cooperative game theory. Unlike traditional feature selection methods, which often rely on global importance scores and may obscure the contributions of features in specific cases, SHAP offers sample-level interpretability. This approach allows us to capture the individual impact of each feature across cases, making it possible to distinguish not only which features are important on average, but also their roles in each unique prediction. In addition, unlike feature selection, SHAP allows us to understand how which levels (high or low) of each biomarker candidate affect disease risk. Such a level of granularity is important in biomedical research, where understanding the distinct impact of biomarkers can be crucial for patient-specific predictions and personalized treatment strategies.

Predictive models were built based on the original dataset (without feature selection/biomarker discovery based on SHAP values) and then predictive models were developed using these candidate markers after feature selection based on SHAP values of predictive models and evaluated in terms of sensitivity, recall and specificity. After the feature selection/biomarker discovery process based on SHAP values, the performance of ML models increased significantly.

After biomarker discovery, the results of the optimal prediction model (LR classifier after biomarker discovery based on SVM-SHAP) that we integrated with SHAP, an XAI approach, identified leucine, isoleucine, L-alloisoleucine, norleucine, and homoserine acids as potential biomarkers for the early diagnosis of BC, and ML models based on these metabolites showed outstanding performance. These identified markers may not only shed light on BC metabolism, but may also lead to the development of new diagnostic approaches. When we combined these metabolite markers, the precision, recall, and specificity for BC classification were 89.50%, 88.38%, and 83.67%, respectively.

Speers et al. reported that maternal embryonic leucine zipper kinase (MELK) ex-pression was significantly higher in breast cancer tissues compared to normal tissue and in TNBC compared to non-TNBC tissue, and the authors reported that MELK RNA and protein expression significantly correlated with radioresistance in BC cell lines (Xie et al., 2023). Another study reported that leucine deprivation inhibited cell proliferation and induced apoptosis of MDA-MB-231 and MCF-7 BC cells (Xiao et al., 2016). Singh et al. reported that leucine restriction was not sufficient to inhibit mammalian target of rapamycin (mTOR) signaling in most BC cell lines, but was associated with activation of the survival molecule Akt, making leucine deprivation an undesirable approach for BC therapy (Singh et al., 2011). In a biomarker discovery study, the amino acid Isoleucine (AUROC ≥ 0.85) was identified as a suitable candidate marker to diagnose and predict BC progression (Eniu et al., 2019).

The content of several intracellular branched-chain amino acids (BCAAs), including valine, leucine and isoleucine, has been reported to decrease after oridonin treatment. The results of this study suggest that oridonin has potent anti-tumor activity *in vitro* and *in vivo* and has potential as an adjuvant to BC treatment regimens. BCAAs are essential amino acids for the human body, and tumor tissues take up BCAAs from surrounding tissues or the bloodstream (Neinast et al., 2019). Therefore, high plasma levels of BCAAs have been as-sociated with cancer development (Mayers et al., 2014). Branched-chain amino acid transaminase 1 (BCAT1), which catalyzes the catabolism of essential BCAAs such as leucine, isoleucine and valine, plays an important role in amino acid metabolism. It can convert BCAAs into the corresponding branched-chain α-keto acids and then into α-ketoglutarate by transferring amino acids to produce glutamic acid, which can further promote the growth of BC cells (Zhang and Han, 2017). These branched-chain keto acids can be further oxidized to form acetyl-CoA and/or succinyl-CoA to feed the TCA cycle or contribute substrates to fatty acid synthesis (Mossmann et al., 2018). In a study that found that intracellular BCAT1 expression was significantly reduced after oridonin treatment, it was reported that this may have reduced intracellular nutrient and energy production. Since amino acids, including BCAAs, can also act as upstream regulators of mTOR activity (Jung et al., 2021), the authors also examined proteins involved in the mTOR signaling pathway. Western blot analysis revealed that the expression of mTOR, PI3K and AKT decreased significantly after oridonin treatment.

The biomarkers identified in the current study in BC management may not only facilitate treatment selection but also play an important role in clinical decision-making processes. Additionally, these molecular signatures may provide clinicians with a nuanced understanding of the heterogeneous nature of BC, allowing them to precisely tailor therapeutic interventions. Moreover, the biomarker-driven prediction model proposed in this study may be promising in determining prognosis by enabling the classification of patients into risk categories and optimizing therapeutic strategies. Consequently, inte-gration of biomarker profiling into routine clinical practice may be beneficial in the personalized management of BC and is important in targeted therapies and improved patient outcomes.

## 5 Limitations

The primary limitation of this study is the lack of an independent validation cohort, which limits the external validity and generalizability of the developed predictive models. Although we used nested cross-validation to address the lack of an external test set and minimize the risk of overfitting, an independent validation in a multicenter setting is important to confirm the applicability of the model to larger populations. Future studies that include larger and more diverse cohorts from multiple institutions may provide a stronger basis for evaluating and improving the performance of the model in different clinical settings. Moreover, the models developed in this study primarily classify BC patients according to their metabolomic profiles, which is consistent with the primary aim of the study to accurately predict BC through the identification of metabolomic biomarkers. Subsequent studies may benefit from integrating patients’ clinical data and incorporating multi-omics information to improve the predictive performance of the models.

## 6 Conclusion

In biomarker discovery and prognostic prediction models, XAI plays an important role in the fight against cancer. The application of XAI applications in medicine has provided a great advantage in obtaining fast and accurate diagnostic results and comprehensive treatment planning. The findings of this study demonstrated the usefulness of the targeted LC-MS/MS analysis-based method for the discovery of BC biomarkers and underlined the importance of hybrid interpretable prediction models combining ML and XAI. In addition, it was determined that SHAP explanations allowed obtaining clinical interpretations of the optimal model with the highest performance in distinguishing BC and understanding the effect of biomarker metabolites, which are the input of the model, on BC.

## Data Availability

The original contributions presented in the study are included in the article/Supplementary Material, further inquiries can be directed to the corresponding authors.
