# Supplementary material for: Biomarker discovery and development of prognostic prediction model using metabolomic panel in breast cancer patients: a hybrid methodology integrating machine learning and explainable artificial intelligence
Source: Front Mol Biosci. 2024 Dec 18;11:1426964. doi: 10.3389/fmolb.2024.1426964 (PMC11688212; doi:10.3389/fmolb.2024.1426964)
Supplement: Supplementary file 1 [file DataSheet1.docx]

**Sample Preparation**

Thawing of frozen samples occurred overnight at 4 °C, with 50 μL of each plasma sample transferred to a 2 mL Eppendorf vial. Protein precipitation and metabolite extraction were conducted by adding 300 μL of methanol. After vortexing for 2 min, the mixture was stored at -20 °C for 30 min, followed by sonication in an ice bath for 10 min and subsequent centrifugation at 14,000 RPM for 20 min at 4 °C. The resulting supernatant (150 μL) was collected into a fresh Eppendorf vial and dried using a Vacufuge Plus evaporator. The dried samples were then reconstituted in 500 μL of 5 mM ammonium acetate in 40% H2O/60% ACN + 0.2% acetic acid containing 5.13 μM l-tyrosine-13C2 and 22.5 μM sodium-l-lactate-13C2 as stable isotope-labeled internal standards to monitor system performance. Additionally, a pooled sample, comprising plasma from all breast cancer (BC) patients and healthy controls, underwent extraction using the same aforementioned procedure. This pooled sample served for quality control (QC) purposes and was analyzed once every 10 study samples.

**Liquid chromatography and mass spectrometry conditions**

The LC-MS/MS method employed in this study was adapted from protocols established and utilized in a growing body of literature [1-6]. In brief, LC-MS/MS experiments were conducted using a Waters Acquity I-Class UPLC TQS-micro MS system (Milford, MA). Each sample underwent dual injections, with 2 μL and 5 μL utilized for analysis in positive and negative ionization modes, respectively. Chromatographic separation was achieved on a Waters Xbridge BEH Amide column (2.5 μm, 2.1 × 150 mm) at 40 °C, with a flow rate of 0.3 mL/min. In positive mode, the mobile phase comprised Solvents A (5 mM ammonium acetate in H2O with 0.1% acetic acid) and B (ACN with 0.1% acetic acid), while in negative mode, Solvent A consisted of 10 mM ammonium bicarbonate in H2O and Solvent B was ACN. The gradient elution conditions remained consistent for both positive and negative ionization modes. Following a 1.5 min isocratic elution of 10% Solvent A, the percentage of Solvent A was linearly increased to 65% at t = 9 min. This composition was maintained for 5 min (t = 14 min), after which it was decreased to 10% at t = 15 min to prepare for subsequent injections. Each injection had a total experimental duration of 30 min. Metabolite identities were confirmed by spiking standard compound mixtures into prepared plasma samples. Integration of extracted MRM peaks was performed using the TargetLynx software (Waters, Milford, MA).

References

1. Jové, M., Collado, R., Quiles, J. L., Ramírez-Tortosa, M. C., Sol, J., Ruiz-Sanjuan, M., ... & Pamplona, R. (2017). A plasma metabolomic signature discloses human breast cancer. *Oncotarget*, *8*(12), 19522.
2. More, T. H., RoyChoudhury, S., Christie, J., Taunk, K., Mane, A., Santra, M. K., ... & Rapole, S. (2018). Metabolomic alterations in invasive ductal carcinoma of breast: A comprehensive metabolomic study using tissue and serum samples. *Oncotarget*, *9*(2), 2678.
3. Zhu, J., Djukovic, D., Deng, L., Gu, H., Himmati, F., Chiorean, E. G., & Raftery, D. (2014). Colorectal cancer detection using targeted serum metabolic profiling. *Journal of proteome research*, *13*(9), 4120-4130.
4. Carroll, P. A., Diolaiti, D., McFerrin, L., Gu, H., Djukovic, D., Du, J., ... & Eisenman, R. N. (2015). Deregulated Myc requires MondoA/Mlx for metabolic reprogramming and tumorigenesis. *Cancer cell*, *27*(2), 271-285.
5. Sperber, H., Mathieu, J., Wang, Y., Ferreccio, A., Hesson, J., Xu, Z., ... & Ruohola-Baker, H. (2015). The metabolome regulates the epigenetic landscape during naive-to-primed human embryonic stem cell transition. *Nature cell biology*, *17*(12), 1523-1535.
6. Gu, H., Zhang, P., Zhu, J., & Raftery, D. (2015). Globally optimized targeted mass spectrometry: reliable metabolomics analysis with broad coverage. *Analytical chemistry*, *87*(24), 12355-12362.
